# Distinct Clinic-Pathological Features of Early Differentiated-Type Gastric Cancers after* Helicobacter pylori* Eradication

**DOI:** 10.1155/2016/8230815

**Published:** 2016-04-26

**Authors:** Noriyuki Horiguchi, Tomomitsu Tahara, Tomohiko Kawamura, Masaaki Okubo, Takamitsu Ishizuka, Yoshihito Nakagawa, Mitsuo Nagasaka, Tomoyuki Shibata, Naoki Ohmiya

**Affiliations:** Department of Gastroenterology, Fujita Health University School of Medicine, Toyoake 470-1192, Japan

## Abstract

*Background*. Gastric cancer is discovered even after successful eradication of* H. pylori*. We investigated clinic pathological features of early gastric cancers after* H. pylori* eradication.* Methods*. 51 early gastric cancers (EGCs) from 44 patients diagnosed after successful* H. pylori* eradication were included as eradication group. The clinic-pathological features were compared with that of 131 EGCs from 120 patients who did not have a history of* H. pylori* eradication (control group).* Results*. Compared with control group, clinic-pathological features of eradication group were characterized as depressed (*p* < 0.0001), reddish (*p* = 0.0001), and smaller (*p* = 0.0095) lesions, which was also confirmed in the comparison of six metachronous lesions diagnosed after initial ESD and subsequent successful* H. pylori* eradication. Prevalence of both SM2 (submucosal invasion greater than 500 *μ*m) and unexpected SM2 cases tended to be higher in eradication group (*p* = 0.077, 0.0867, resp.). Prevalence of inconclusive diagnosis of gastric cancer during pretreatment biopsy was also higher in the same group (26.0% versus 1.6%, *p* < 0.0001).* Conclusions*. Informative clinic pathological features of EGC after* H. pylori* eradication are depressed, reddish appearances, which should be treated as a caution because histological diagnosis of cancerous tissue is sometimes difficult by endoscopic biopsy.

## 1. Introduction

It is well accepted that* Helicobacter pylori* (*H. pylori*) infection is closely associated with the development of gastric cancer based on the epidemiological [1–3] and experimental studies [4, 5]. A prospective study demonstrated that* H. pylori* eradication therapy reduces incidence of metachronous gastric cancer following endoscopic resection of early gastric cancer [6]. Therefore, Japanese National Health Insurance System has recently covered the cost for the eradication therapy for patients with* H. pylori*-associated gastritis. However, it has been realized that considerable percentage of patients develop gastric cancer even after successful* H. pylori* eradication [7]. Moreover, it is possible that* H. pylori* eradication influences clinic-pathological features of gastric tumors, including macro- and microscopic appearances [8–10]. Accordingly, we investigated clinic-pathological features of early gastric cancer (EGC) with or without history of prior* H. pylori* eradication therapy.

Our data demonstrated several distinct clinic-pathological features of patients with EGC after successful* H. pylori* eradication, providing helpful information for the better clinical implementation of these patients.

## 2. Patients and Methods

### 2.1. Study Population

We studied 51 EGCs from 44 patients diagnosed at least 6 months after the successful* H. pylori* eradication therapy for the various reasons, including gastric and duodenal ulcer or scaring and chronic gastritis, or after endoscopic resection of early gastric cancer (eradication group). Median period after eradication therapy was 36 months (ranged between 6 to 180 months). We also included 131 EGCs from 120 patients who did not have a history of* H. pylori* eradication therapy as the control group. All patients attended the endoscopy center of Fujita Health University for the ESD between April 2007 and April 2015. ESD was performed for 171 lesions, while 11 lesions were considered to be surgical indication during initial endoscopic examination and laparoscopy-assisted total gastrectomy was performed for these cases. Fujita Health University School of Medicine approved the protocol, and written informed consent was obtained from all participating subjects.

### 2.2. Evaluation of* H. pylori* Status

Evaluation of* H. pylori* eradication treatment in the eradication group was based on the urea breath test as well as the histological assessment using endoscopic biopsy specimens obtained from nonpathological mucosa of the greater curvature of gastric antrum and upper corpus. If the results were negative in both examinations, we considered that* H. pylori *eradication had been successfully performed. 104 patients from control groups were* H. pylori* positive based on the either 3C-urea breath test, serum titer, or histological assessment, while the remaining patients were negative for all above clinical tests for* H. pylori* infection but have no history of* H. pylori* eradication therapy. Six lesions from 5 patients in the eradication group were metachronous cases from the control group who were diagnosed after initial ESD and subsequent successful* H. pylori *eradication.

### 2.3. Clinic-Pathological Characteristics of EGCs

In both groups, age and sex were investigated based on the medical record.

Anatomical location and color were investigated based on the endoscopic image obtained during the esophagogastroduodenoscopy (EGD) examination before ESD. Histological assessment of resected specimens was performed and all lesions were diagnosed as differentiated adenocarcinoma. Morphologic appearance was classified as either protruding (0-I, 0-IIa) or depressed (0-IIc, 0-IIa + IIc, and 0-IIc + IIa), and submucosal invasion was also defined as SM1 (cases with submucosal invasion less than 500 *μ*m) and SM2 (cases with submucosal invasion greater than 500 *μ*m) according to the Japanese Classification of Gastric Carcinoma, 14th edition [11]. We also investigated the prevalence of SM2 cases from the patients performing ESD, being considered to be the indication of ESD. We defined these SM2 cases as unexpected SM2. Moreover, we reviewed the result of the targeted biopsy from the lesion during the pretreatment EGD. Prevalence of inconclusive diagnosis of gastric cancer from the targeted biopsy was investigated. The definition of inconclusive diagnosis of gastric cancer is material for which diagnosis of neoplastic or nonneoplastic lesion is difficult despite sufficient amount of tissues. This result was based on the report from senior pathologists in our hospital.

### 2.4. Statistical Analysis

Continuous variables between two groups were determined using the Mann-Whitney *U* test. Categorical variables were determined using the chi squared test. Differences at *p* values less than 0.05 were considered to be statistically significant.

## 3. Results 

### 3.1. Distinct Clinic-Pathological Features of EGCs Diagnosed after* H. pylori* Eradication Therapy

Table 1 indicates clinic-pathological characteristics among eradication and control groups. Although age, male sex, and anatomical location were not significantly different among those two groups, prevalence of depressed (88.2% versus 57.3%, *p* < 0.0001) and reddish lesions (88.2% versus 54.9%, *p* = 0.0001) was significantly higher in eradication group.

We also found that tumor size in eradication group was significantly smaller than that of control group (11.7 ± 1.0 mm versus 13.5 ± 0.6 mm, *p* = 0.0095). On the other hand, prevalence of both SM2 and unexpected SM2 cases tended to be higher in eradication group (*p* = 0.077, 0.0867, resp.) (Table 1). When the size of SM2 lesions was compared among the two groups, it was also found that SM2 lesions from eradication groups were significantly smaller (*p* = 0.008, Figure 2). Since six lesions from 5 patients in the eradication group were metachronous cases from the control group, being diagnosed after initial ESD and subsequent successful* H. pylori* eradication, we compared clinic-pathological features of initial and metachronous lesions (Table 2). The initial lesions consisted of five protruding (0-IIa or 0-I) lesions and the three of these were reddish colors. On the other hand, the metachronous lesions appeared as depressed (0-IIc, five out of six lesions) and reddish (all) colors. It was also shown that metachronous lesions were of smaller size compared to the initial lesions (five out of six lesions) (Table 2 and Figure 3).

We also investigated prevalence of inconclusive diagnosis of gastric cancer from the targeted biopsy during the pretreatment EGD. This analysis was done for 50 lesions from eradication group and 127 lesions from control group whose targeted biopsy samples were sufficient for histological assessment (Table 3). It was shown that the prevalence of the inconclusive diagnosis was significantly higher in the eradication group than in control group (26.0% versus 1.6%, *p* < 0.0001). For all these lesions, diagnosis of gastric cancer was finally confirmed by the resected specimens.

## 4. Discussion

Previous report described the features of gastric cancer detected after successful* H. pylori *eradication as a lesion less than 20 mm in size, located in the middle and lower anatomical location, a depressed morphological type [7], which was also confirmed in the recent study [10]. In the present study, EGCs in the eradication group were mainly less than 20 mm (11.7 ± 1.0 mm) in size, which was significantly smaller than that of control group. Morphologically, the prevalence of depressed type was also significantly higher in the same group. On the other hand, although about 80% of lesions appeared in the middle and lower anatomical location, prevalence of the cases located in the upper anatomical location seemed rather higher in the eradication group when compared to control group (21.6% versus 10.7%), which suggests that considerable gastric cancer cases also arise in the upper third in the stomach after successful* H. pylori* eradication. We have also shown that one informative endoscopic feature is reddish appearance, not noted in other reports [7, 10]. Therefore, based on our result, informative morphological feature in the eradication group would be depressed, reddish, and tiny lesions. These characteristics were also confirmed in the comparison of metachronous cases diagnosed after initial ESD and subsequent successful* H. pylori* eradication. Concerning the morphological change in gastric cancer after successful* H. pylori* eradication, Ito et al. have reported that the endoscopic features of gastric cancer changed to a flattened and indistinct form after* H. pylori* eradication. Notably, these changes were observed after a short term 1-month follow-up [8]. In this study, prevalence of depressed, reddish, and tiny lesions did not differ regardless of their periods after eradication (data not shown). Moreover, three cases all were diagnosed within one year after eradication, which might have been developed before eradication, presented depressed, reddish, and tiny lesions (less than 13 mm). This suggests that* H. pylori* eradication would influence the morphological appearance within a short term, which should be noted for the diagnosed EGC in patients after* H. pylori* eradication.

**Figure 1:**
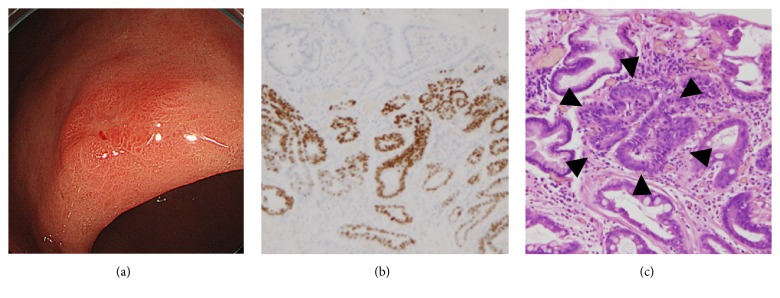
A typical case of EGC diagnosed after successful* H. pylori* eradication treatment. EGD indicated a depressed (0-IIc), reddish lesion in the lesser curvature of the gastric corpus (a). Diagnosis of neoplastic or nonneoplastic lesion was difficult by the targeted biopsy during the pretreatment EGD. Histological assessment of resected specimen showed nonneoplastic epithelium covering the cancerous tissue, (b) and (c). (b) TP53 immunohistochemistry; (c) hematoxylin and eosin stain. Cancer crypts were indicated by the black arrowhead.

**Figure 2:**
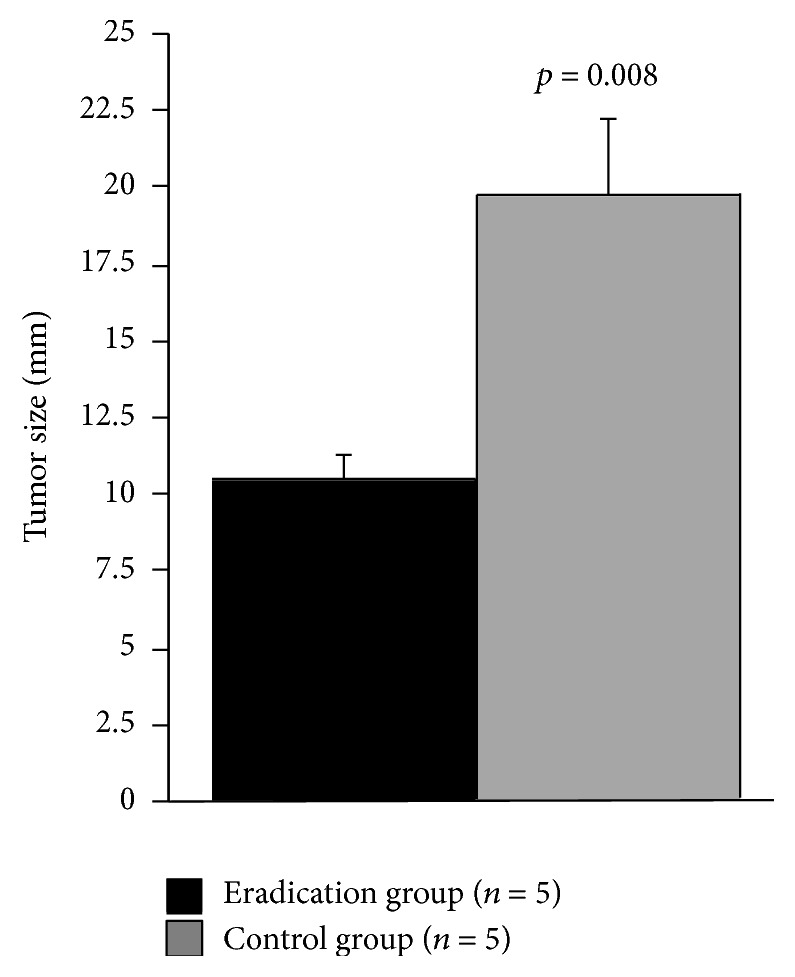
Comparison of lesion size of SM2 lesions among eradication and control groups. Statistical analysis was performed using the Mann-Whitney *U* test.

**Figure 3:**
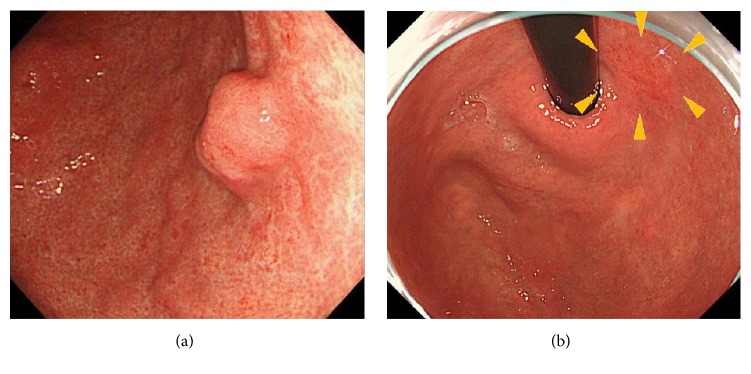
A metachronous lesion. A reddish depressed (type 0-IIc) lesion (b: yellow arrowhead) was detected after 60 months of initial ESD and subsequent* H. pylori *eradication for the initial lesion (a).

We have shown that the prevalence of SM2 lesions was rather higher in the eradication group despite its tiny size. Moreover, unexpected SM2 cases also tended to be higher in the same group, possibly because the tumor size of SM2 lesions was also smaller in this group. This should be treated as a caution that patients who have history of* H. pylori *eradication potentially develop EGC in tiny and indistinct form, but considerable percentage of them includes SM2 cases. Recent study demonstrated that the feature of histopathology of eradication case is normal columnar epithelium over the neoplasm [8, 10], while histological surface differentiation has also been reported as another feature [9]. For these histological characteristics, endoscopic diagnosis of EGC after eradication often becomes difficult due to disappearance of cancerous characteristics using the magnifying NBI endoscopy [9, 10]. We have also shown that inconclusive diagnosis of gastric cancer from the targeted biopsy was significantly frequent (26%) in eradication group. Since eradication group has either normal epithelium (Figure 1(c)) or surface differentiation covering over the tumor tissue, it is possible that diagnosis of neoplastic or nonneoplastic lesion would become difficult despite sufficient amount of tissues. Previous report suggested that the risk of gastric cancer development after successful* H. pylori *eradication would be a baseline severe atrophic gastritis in the corpus [7]. Therefore, informative endoscopic appearances, such as depressed, reddish lesions should be carefully followed up in such patients even if its biopsy result was negative.

Recent development of sequencing analysis of gastric microbiota showed that* H. pylori* was not alone and the interaction of* H. pylori* with those microorganisms might play a part in* H. pylori*-associated gastric carcinogenesis [12]. It is also proposed that the effectiveness of* H. pylori *eradication on gastric cancer incidence may partially be attributed to* H. pylori *eradication role in inhibiting non-*H. pylori* residents in the stomach [13]. However, no convincing study has reported the effect of* H. pylori *eradication on non-*H. pylori* gastric microbiota so far. Future study will be also needed to clarify the gastric cancer risk after* H. pylori *eradication from the different view point such as gastric microbiota beyond* H. pylori.*

## 5. Conclusion

Informative clinic-pathological features of EGC after* H. pylori* eradication are depressed, reddish appearances, which should be treated as a caution because histological diagnosis of cancerous tissue is sometimes difficult by endoscopic biopsy.

## Figures and Tables

**Table 1:** Clinic-pathological characteristics of EGC lesions among eradication and control groups.

Variables	Eradication group	Control group	*p* value
	(51 lesions)	(131 lesions)	
Median age (range)	74 (53–89)	72 (53–92)	0.29
Male % (*n*)	74.5% (38)	81.7% (107)	0.28
Location: U/M/L % (*n*)	21.6%/41.2%/37.2%	10.7%/40.5%/48.8%	0.12
	(11/21/19)	(14/53/64)	
Morphology^*∗*^: protruding/depressed % (*n*)	11.2%/88.2%	42.7%/57.3%	<0.0001
	(6/45)	(56/75)	
Color: redness/whiteness/same as surroundings % (*n*)	88.2%/7.8%/3.9%	54.9%/28.2%/16.7%	0.0001
	(45/4/2)	(72/37/22)	
Tumor size: ±SE	11.7 ± 1.0 mm	13.5 ± 0.6 mm	0.0095
Depth^*∗∗*^: M/SM1/SM2 % (*n*)	90.1%/0%/9.9%	90.8%/5.3%/3.8%	0.077
	(46/0/5)	(119/7/5)	
Unexpected SM2 case^*∗∗∗*^	8.7% (4/46)	2.4% (3/124)	0.0867

^*∗*^Protruding, 0-I and 0-IIa; depressed, 0-IIc, 0-IIa + IIc, and 0-IIc + IIa according to the Japanese classification.

^*∗∗*^SM1, cases with submucosal invasion less than 500 *μ*m; SM2, cases with submucosal invasion greater than 500 *μ*m.

^*∗∗∗*^Patients whose SM2 invasion could not be expected before ESD.

Age and tumor size were compared using Mann-Whitney *U* test.

Location, morphology, color, depth, and unexpected SM2 case were compared using chi statistics.

**Table 2:** Six metachronous cases diagnosed after initial ESD and subsequent successful eradication.

Case number	Period after eradication (months)	Age	Gender	Morphology	Location	Depth	Size (mm)	Color
Case 1^*∗*^	18	78	M	0-IIa → 0-IIa	U → U	M → M	35 → 15	Same as surroundings → redness
Case 2^*∗*^	24	78	M	0-IIa → 0-IIc	U → L	M → M	35 → 10	Same as surroundings → redness
Case 3	60	75	M	0-IIc → 0-IIc	M → U	M → SM2	15 → 8	Redness → redness
Case 4	32	89	F	0-I → 0-IIc	M → U	M → M	15 → 5	Same as surroundings → redness
Case 5	72	82	M	0-I → 0-IIc	L → L	M → M	10 → 3	Redness → redness
Case 6	60	76	F	0-I → 0-IIc	L → L	M → M	8 → 20	Redness → redness

^*∗*^Cases 1 and 2 are from the same patient.

**Table 3:** Prevalence of inconclusive diagnosis of gastric cancer by initial endoscopic biopsy.

	Eradication group (*n* = 50)	Control group (*n* = 127)	*p* value
Inconclusive diagnosis by initial endoscopic biopsy % (*n*)	26.0% (13/50)	1.6% (2/127)	<0.0001

Note: we excluded four patients from control group whose biopsy samples were tiny for histological analysis.

One patient in the eradication group did not perform biopsy before ESD.
